# Integrated *de novo* Analysis of Transcriptional and Metabolic Variations in Salt-Treated *Solenostemma argel* Desert Plants

**DOI:** 10.3389/fpls.2021.744699

**Published:** 2021-11-19

**Authors:** Hasan Ahmad, Mohamed Maher, Eslam M. Abdel-Salam, Yufei Li, Chenkun Yang, Nagwa ElSafty, Mohamed Ewas, Elsayed Nishawy, Jie Luo

**Affiliations:** ^1^National Key Laboratory of Crop Genetic Improvement and National Center of Plant Gene Research (Wuhan), Huazhong Agricultural University, Wuhan, China; ^2^National Gene Bank, Agricultural Research Center, Giza, Egypt; ^3^Biochemistry Department, Faculty of Agriculture, Zagazig University, Zagazig, Egypt; ^4^Department of Botany and Microbiology, College of Science, King Saud University, Riyadh, Saudi Arabia; ^5^Plant Genetics Resources Department, Desert Research Center, Cairo, Egypt; ^6^College of Tropical Crops, Hainan University, Haikou, China

**Keywords:** transcriptome, metabolome, phenylpropanoid, salt stress, *Solenostemma argel*

## Abstract

*Solenostemma argel* (Delile) Hayne is a desert plant that survives harsh environmental conditions with several vital medicinal properties. Salt stress is a major constraint limiting agricultural production around the globe. However, response mechanisms behind the adaptation of *S. argel* plants to salt stress are still poorly understood. In the current study, we applied an omics approach to explore how this plant adapts to salt stress by integrating transcriptomic and metabolomic changes in the roots and leaves of *S. argel* plants under salt stress. *De novo* assembly of transcriptome produced 57,796 unigenes represented by 165,147 transcripts/isoforms. A total of 730 differentially expressed genes (DEGs) were identified in the roots (396 and 334 were up- and down-regulated, respectively). In the leaves, 927 DEGs were identified (601 and 326 were up- and down-regulated, respectively). Gene ontology and Kyoto Encyclopedia of Genes And Genomes pathway enrichment analyses revealed that several defense-related biological processes, such as response to osmotic and oxidative stress, hormonal signal transduction, mitogen-activated protein kinase signaling, and phenylpropanoid biosynthesis pathways are the potential mechanisms involved in the tolerance of *S. argel* plants to salt stress. Furthermore, liquid chromatography-tandem mass spectrometry was used to detect the metabolic variations of the leaves and roots of *S. argel* under control and salt stress. 45 and 56 critical metabolites showed changes in their levels in the stressed roots and leaves, respectively; there were 20 metabolites in common between the roots and leaves. Differentially accumulated metabolites included amino acids, polyamines, hydroxycinnamic acids, monolignols, flavonoids, and saccharides that improve antioxidant ability and osmotic adjustment of *S. argel* plants under salt stress. The results present insights into potential salt response mechanisms in *S. argel* desert plants and increase the knowledge in order to generate more tolerant crops to salt stress.

## Introduction

Various environmental factors, such as salt, drought, and high temperatures limit the food production, worldwide (Benjamin et al., 2019). Recently, global climate changes lead to major effects on soil, which change the characteristics of the soil in different parts of the world. These effects include soil salinization resulting from decreased precipitation, increased temperature, and drought, causing the accumulation of different salts, especially sodium chloride, in the top layers of the soil (Okur and Örçen, 2020). Salt restricts the growth and yield of plants. In general, there are two harmful effects of NaCl on plants: (i) reducing the supply of water to plants from the soil by roots (osmotic stress) and (ii) toxic effects of plants accumulate Na^+^ and Cl^–^ ions (ionic stress; van Zelm et al., 2020).

Although, the sodium sensing mechanism of the plant is yet to be determined, once high sodium levels in the soil occur intra- or extracellularly, sodium sensing occurs (the initial perception). After that, early responses, such as the generation of reactive oxygen species (ROS), Ca^2+^ signaling, and the transport of K^+^ and H^+^ to reduce sodium import into the plant occur. This early signaling phase follows the change of phytohormone levels, and the gene expression levels end with adaptive responses to salt, e.g., the production of compatible osmolytes including charged metabolites (proline and polyamine), soluble sugars (fructose and sucrose), polyols (glycerol, mannitol, and sorbitol), and complex sugars (trehalose) for osmotic adjustment and ROS scavenging increases turgor and expansion of cells to growth and development (Yang and Guo, 2018; Isayenkov and Maathuis, 2019; van Zelm et al., 2020).

Moreover, the metabolisms of amino acids, starch and sucrose, and phenylpropanoid biosynthesis pathways are linked to salt tolerance (Gan et al., 2021). In plants, the phenylpropanoid pathway is responsible for the biosynthesis of many secondary metabolites; both flavonoids and lignins are synthesized that rebalance the cellular ROS and have a vital role in plant defense under abiotic stresses. All these changes and more happen inside the plant to adapt to environmental changes (Rossi et al., 2016; Yang et al., 2017; Zhang et al., 2017).

*Solenostemma argel* (Delile) Hayne is a herbaceous ornamental plant commonly cultivated in Africa and in the deserts of the Middle East. It is the only known species in the genus, *Solenostemma*, subfamily Asclepiadoideae, and family, Apocynaceae. In traditional herbal medicine, *S. argel* plants are used to enhance immunity, support kidney and liver functions, and to treat anti-urinary tract infections; when used as an anti-rheumatic agent, it greatly increased all histopathological parameters (Al-Juhaimi et al., 2018; El-Shiekh et al., 2021), such as flavones (kaempferol, isorhamnetin, naringenin, and quercetin), phenols (*trans*-cinnamic acid, gallic acid, caffeic acid, and syringic acid), polyphenols (resveratrol and catechol), glycosylated flavonoids (apigenin-7-glucoside and quercetin-3-rutinoside), pregnane, pregnenes, β-sitosterol, and β-carotene (Plaza et al., 2005; Ounaissia et al., 2016).

Fortunately, *S. argel* plants can tolerate extreme climatic conditions in arid and semi-arid settings. Therefore, *S. argel* is known as a desert herb. Previous studies indicated that some milkweeds, such as *Cynanchum auriculatum*, *Cynanchum acutum*, and *Cynanchum chinense* have demonstrated tolerance to salt, and they are the closest member of milkweeds to *S. argel* plants (Mercado et al., 2012; Sharawy, 2013; Zhang M. et al., 2020). However, no study has been performed on *S. argel* plants to find how they react to abiotic stress at the molecular stage.

Integrating multi-omics results, such as techniques based on transcriptomic and metabolomic studies under environmental stress, has recently become a familiar and successful method for improving our understanding of abiotic stress tolerance (Haider et al., 2017; Wang et al., 2019; Szepesi, 2020). Several genetic and metabolic networks involved in the mechanism of salt tolerance have been studied in plants. However, the genetics behind salt stress tolerance in plants is still unclear and needs more elaboration by examining transcriptomic and metabolomic regulation changes in either sensitive or tolerant plants under salt stress conditions (Luo, 2015; Guan et al., 2018; Yang and Guo, 2018).

The plants in arid and semi-arid regions that are exposed to high levels of various abiotic stresses, especially salt and drought might provide an ideal model to understand genes and metabolites that play critical roles in response to abiotic stress. Furthermore, concerning strategies to enhance the tolerance of crops, these plants may provide valuable insights into salt tolerance mechanisms (Gu J. et al., 2018; Benjamin et al., 2019). Thus, the current study would use combined transcriptomic and metabolomic evidence to analyze improvements in the roots and leaves of *S. argel* desert plants triggered by soil salt stress, which were poorly understood before. The results obtained in the current study would lay the foundation for future biotechnological studies examining the possible salinity tolerance mechanisms in *S. argel* plants.

## Materials and Methods

### Plant Growth and Stress Treatment

Seeds of *S. argel* plants were collected from a saline field in Siwa Oasis (29°12′13.0″N 25°31′09.5″E), Egypt. Before cultivation, the seeds were soaked in tap water for 3 days. Germinated seeds were then transferred to 10-cm-diameter pots containing clay soil and maintained in a greenhouse under 22 ± 2°C with artificial illumination for 16/8 day/night photoperiod and light intensity of 1,600 mol m^–2^ s^–1^. To apply salt stress, the pots were randomly divided into two groups: control and salt-stressed groups. Each group contained ten replicates (pots) with five plants in each pot. Each pot was irrigated every 4 days with a fixed amount (200 ml) of tap water for the control group or 500 mM NaCl solution for stress treatment. Salt stress treatment started after 30 days of seed cultivation and continued for 3 days before the collection of samples.

### Total RNA Extraction

Total RNA was extracted from the leaves and roots of two biological replicates of *S. argel* plants either under control or stress conditions after 3 days of salinity treatment. RNA was extracted using TRIzol^TM^ Reagent (Invitrogen Corporation, MA, United States) following the protocol of the manufacturer. Quantity and quality of the extracted RNA were checked using (1) Cale K5500 spectral luminometer (KO, Beijing, China) to measure the purity of RNAs, (2) 1% agarose gel electrophoresis to detect degradation and impurities, and (3) Agilent 2100 RNA Nano 6000 Assay Kit (Agilent Technologies, CA, United States) to examine the integrity and concentration of RNA.

### Library Construction and RNA Sequencing

Complementary DNA (cDNA) libraries for all samples were constructed using 3 μl of total qualified RNA extracted from each sample. The messenger RNA (mRNA) was enriched in each library *via* Dynabeads^®^ Oligo (dT) magnetic beads (ThermoFisher Scientific, CA, United States). Subsequently, a fragmentation buffer was added to the mRNA to obtain short fragments. These mRNA short fragments were used as a template with a random hexamer primer to synthesize the first strand of cDNA. NEBNext^®^ Ultra^TM^ RNA Library Prep Kit for Illumina^®^ (NEB, MA, United States) containing a buffer (NEBNext Second Strand Synthesis Reaction Buffer), dNTPs, RNaseH, and DNA polymerase was used to synthesize the cDNA double-strand (ds-cDNA or cDNA library) according to the protocol of the manufacturer. The ds-cDNA was purified by the QIAquick PCR Purification Kit (Qiagen Inc., MD, United States). Afterward, the ds-cDNA was eluted with EB buffer and then subjected to perform end repair, dA-tailing, and adapter-ligation.

Finally, the target size fragments were recovered by agarose gel electrophoresis and PCR amplification to complete the entire library preparation. The PCR product was purified using Agencourt AMPure XP Beads (Agencourt Bioscience Corporation, MA, United States) and the library quality was assessed on a Bioanalyzer (Agilent High Sensitivity Chip; Agilent Technologies Inc., CA, United States). Finally, the constructed library was sequenced using the Illumina platform, and the sequencing strategy was done by paired-end 150 bp (PE 150).

### Data Analysis

#### Data Quality Control

The original base call sequencing of the BCL files of Illumina (CA, United States) were demultiplexed into FASTQ files using the Illumina bcl2fastq2 Conversion Software v2.20. The quality of the obtained raw reads was examined using FastQC software v0.11.9. All low-quality reads, including reads containing adapters, with more than 5% N (base unknown) bases, and with Q ≤ 19 bases account for 50% of the total bases, were removed using Trimmomatic software v0.32 (Williams et al., 2016). Read pairs were dropped even if one read did not pass the quality matrices. Obtained clean reads were used for further analysis.

#### *De novo* Transcriptome Assembly

Full-length transcripts of *S. argel* plants were assembled using Trinity software v2.4.0 (Grabherr et al., 2011). Using the basic principles of de Bruijn graph theory, the Trinity assembles full-length transcripts based on the characteristics of alternative splicing of transcripts. Based on the transcript sequence, the longest transcript sequence in each gene was considered unigene. First, all assembled trinity (transcripts) and unigenes were stored in fasta files. Next, the length distribution and guanine-cytosine (GC) content of transcripts (trinity and unigene were counted separately) were collected to check the quality of the transcripts.

#### Assembly Quality Control

The quality of the assembled transcriptome was assessed using the statistics provided by Trinity assembler, including GC content, minimum length, maximum length, average length, and N50 length. Furthermore, bowtie2 v2.2.3 (Langmead and Salzberg, 2012) were used to align RNA-generated reads from all samples against the assembled transcriptome and the average mapping percentage was considered a quality indicator. Finally, the presence of Benchmarking Universal Single-Copy Orthologs (BUSCOs) was examined using BUSCO v3.0.1 to verify the completeness of the assembled transcripts (Simão et al., 2015).

#### Transcriptome Functional Annotation

The *de novo* assembled transcriptomes were functionally annotated using Trinotate v3.0.2 (Bryant et al., 2017). Trinotate performs comprehensive annotation of all assembled unigenes *via* translated assembled unigenes into their potential polypeptide chain using TransDecoder and scan all the assembled unigenes and their products using blastx and blast tools against several databases, i.e., Uniprot, eggNOG, Gene ontology (GO), and Kyoto Encyclopedia of Genes And Genomes (KEGG). Moreover, Trinity applies the prediction of the functional roles of assembled unigenes and their products using HMMERSCAN, SignalP, TmHMM, and RNAmmer tools.

### Differential Expression Analysis

#### Quantitation of Gene Expression Levels

Gene expression level is generally measured by the amount of mRNA transcribed by the gene. HTSeq-count tool v0.6.0 was used to count the presence of each gene in each sequenced sample (Anders et al., 2015). Reads per kilobase of transcript per million mapped reads (RPKM) is an effective tool for quantitatively estimating gene expression values using RNA-Seq technology and to eliminate the effect of sequencing depth and gene length on gene expression levels (Mortazavi et al., 2008). Therefore, RPKM was calculated to estimate the expression level of genes in each sample.

#### Identification of Differentially Expressed Genes

Differentially expressed genes (DEGs) were identified in the leaves and roots separately by the comparison of controlled *S. argel* plants with salt-stressed ones. In this regard, DESeq2 package v1.4.5 in R programming language was used to compare the samples in each group (the leaves and the roots) with two biological replicates. DESeq2 uses the negative binomial distribution model and it enables a more accurate analysis of differential expression between the libraries (Love et al., 2014). According to the approach by Benjamini and Hochberg, *P*-value was modified to calculate the false discovery rate for each gene. Only genes with *q* value (adjusted *P*-value) ≤ 0.05 and |log2_ratio| > 1 were recognized as DEGs.

#### Functional Annotation of Differentially Expressed Genes

Identified up- and down-regulated DEGs were used separately to identify the enriched GO terms and KEGG pathways. First, the GO enrichment by up- and down-regulated DEGs was examined using the hypergeometric test *via* Blast2go software with *q* value ≤ 0.05 and |log2_ratio| > 1 (Conesa et al., 2005). Similarly, KEGG pathway enrichment was investigated using KEGG website^1^ with *q* value ≤ 0.05 and |log2_ratio| > 1.

### Real-Time Quantitative Reverse Transcription PCR Validation

The expression levels of 16 DEGs (6 in the roots, 6 in the leaves, and 4 in both the roots and the leaves) involved in metabolic pathways were examined using qRT-PCR to validate their analysis using RNA-Seq data (Maher et al., 2021). The genes were selected to cover all the possible pathways and/or mechanisms identified as involved in the salinity tolerance in the studied plant. Goldenstar^TM^RT6 cDNA Synthesis Mix (gDNA remover and Rnasin selected; TSINGKE, China) was used to construct cRNA molecules from each mRNA. The mRNA molecules were amplified by qRT-PCR using 2 × TSINGKE Master Qpcr Mix (SYBR Green I). Expression of each examined gene was normalized to the reference gene, BnActin7 gene. The relative quantitative expression levels of the DEGs were determined using the 2^–ΔΔCt^ method (Schmittgen and Livak, 2008). The primers used for each gene designed using the Primer3 software are shown in the Supplementary Table 1.

### Metabolomic Profiling

Freeze-dried samples (0.50 *g*) of the root and leaf were collected. After that, the freeze-dried samples were machined with a zirconia grinding ball at 30 Hz for 1.5 min using a mixer mill (MM 400, Retsch, Haan, Germany). A 100 mg sample was collected at 4°C with 1 ml of 100 percent methanol containing 0.1 mg/L of lidocaine for metabolites of lipid solubility or 70:30 methanol: 0.1 mg/L of lidocaine containing water (internal standard) for metabolites of water solubility overnight. Then, for each sample extract, centrifugation was performed at 10,000 *g* for 10 min. CNWBOND Carbon-GCB SPE Cartridge, 250 mg, 3 ml, was used for the solid-phase extraction of lipid-solubility extracts, following the mixing of 0.4 ml of each sample extract and the filtration of the resulting mixture by SCAA-104, 0.22 μm pore size before the study of LC-electrospray ionization (ESI)-MS/MS (Chen et al., 2013). Metabolite quantification was done using a scheduled multiple reaction monitoring (MRM) method (Dresen et al., 2010). An MRM detection window of 80 s and a target scan time of 1.5 s was used (Chen et al., 2014). Finally, only those compounds that were present in 100% of replicates (*N* = 6) within at least one procedure were maintained after postprocessing. All metabolite data were log2-transformed to boost normality.

## Results

### Plant Growth and Transcriptome Assembly and Annotation

The changes in phenotypic characteristics induced by exposing *S*. *argel* plant to salinity stress are shown in Supplementary Figure 1. All the generated reads for all *S. argel* plant samples were pooled together and assembled using Trinity software. The total number of assembled trinity (transcripts/isoforms) reached 165,147 transcripts representing 57,796 unigenes (genes). The total number of assembled bases to produce all isoforms was 275,324,172 bp with 38.46% GC content, while unigenes were constructed using 61,325,868 bp with 37.93% GC. The average transcript length was 1,667.15 bp, while the average unigene length was 1,061.07. Moreover, the N50 of the assembled transcripts were 2,758 bp, but for unigenes, they were 2,128 bp only (Table 1).

**TABLE 1:** Summary statistics of the assembled transcriptome of *S. argel* plants and distribution of the generated sequence lengths.

Basic stat	Trinity	Unigene
Number of assembled sequences	165,147	57,796
Percent GC*	38.46	37.93
Total assembled bases	275,324,172	61,325,868
N50	2,758	2,128
N90	822	385
Min	201	201
Max	17,615	17,615
Mean	1,667.15	1,061.07

*Percent GC indicates the percentage of guanine (G) and cytosine (C) bases in the assembled whole transcriptome of *S. argel*.

### Coding and Non-coding Genes Identification

**FIGURE 1:**
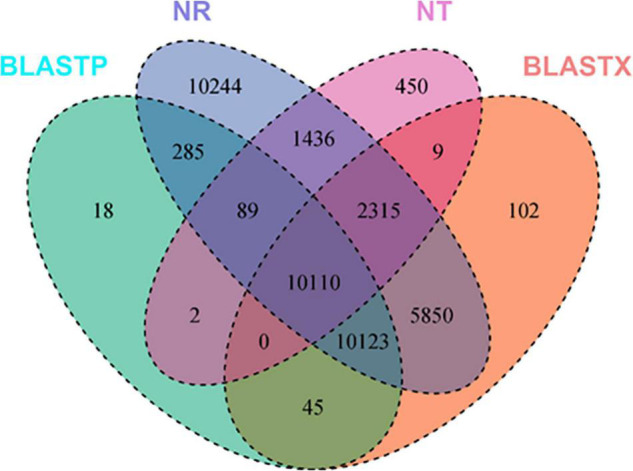
Number of annotated *de novo* assembled unigenes of *S. argel* plants using different tools, e.g., Blastx, Blastp, HmmScan, SignalP, TmHMMP, and RNAmmer against several databases, e.g., UniProt (NT), RefSeq non-redundant (NR) protenis, KEGG Orthology (KO), and gene ontology (GO) term databases.

A total of 57,796 unigenes were annotated after searching all the *de novo* assembled unigenes against different protein and RNA databases using different tools, e.g., Blast, HmmScan, SignalP, and TmHMMP. Blastp and blastx tools were used to search translated peptides and nucleotides against the UniProt database, respectively. All the assembled unigenes were annotated at least in one of the searched databases. Roughly, 70% of the assembled unigenes were annotated using nucleotide sequences against the NR database. Blastx and blastp search against the UniProt database identified 49.4 and 35.8% of the assembled unigenes, respectively. Moreover, 11.31% of the assembled unigenes were found to have transmembrane helices in their protein products. Around 25.54 and 49.14% of the assembled unigenes were functionally characterized in at least one of the KEGG orthology (KO) pathways and GO terms, respectively. Eight (0.01%) genes were annotated as ribosomal RNAs using RNAmmer. Usage of SignalP showed that 3.48% of the assembled unigenes has potential signal peptides. Searching all the assembled unigenes against eggNOG and Pfam databases annotated 18,973 (32.83%) and 20109 (34.79) unigenes, respectively. Several unigenes were annotated in different databases while others were annotated in only one data base. In total, 10,110 sequences were annotated in Ref-Seq, nucleotide, and UniProt databases using either nucleotide sequences or their translated polypeptide chains generated using TransDecoder (Figure 1). Blastp and blastx tools uniquely annotated 18 and 102 unigenes that were not annotated in any other database.

### Identification of Differentially Expressed Genes

**FIGURE 2:**
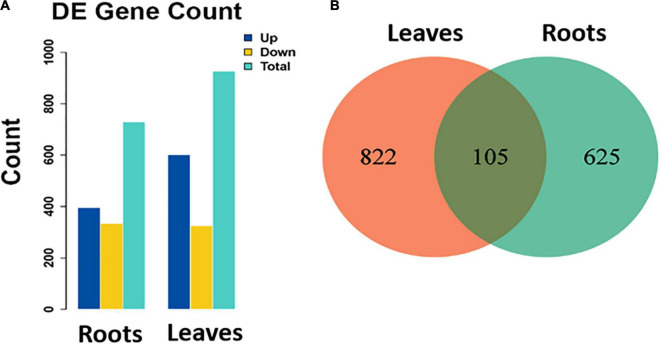
**(A)** Number of identified DEGs (total, up-regulated, and down-regulated) in stressed roots (T1_C1) and leaves (T2_C2) as compared to the control ones of *S. argel* plants and **(B)** a Venn diagram shows the number of common and unique DEGs identified in each comparison.

Samples obtained from the roots under control or stressed conditions were compared together and those obtained from leaves either under control or stressed conditions were also compared together. In the roots, 730 DEGs were identified; out of them, 396 and 334 were up- and down-regulated, respectively, in stressed roots as compared to the control ones (Figure 2A). Similarly, in the leaves, 927 DEGs were identified (601 up-regulated and 326 down-regulated in stressed leaves compared to the control ones). There were 105 DEGs common in between the leaves and the roots (Figure 2B).

**FIGURE 3:**
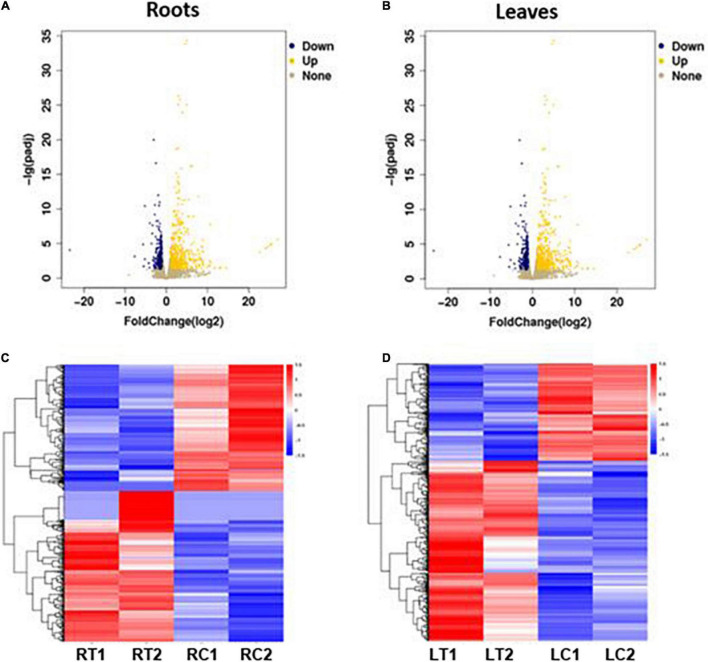
Volcano plots showing the change in expression values among all expressed genes in the roots **(A)** and leaves **(B)** of *S. argel* plants under salt stress as compared to control, and heatmaps showing the expression values of all identified DEGs in the roots **(C)** and leaves **(D)** of these plants. RT1: roots treated with salinity stress replicate 1, RT2: roots treated with salinity stress replicate 2, RC1: control roots replicate 1, RC2: control roots replicate 2, LT1: leaves treated with salinity stress replicate 1, LT2: leaves treated with salinity stress replicate 2, LC1: control leaves replicate 1, and LC2: control leaves replicate 2.

The volcano plots in Figures 3A,B show the changes in the expression of all genes in the roots and leaves of the stressed *S. argel* plants compared to the control plants. A gene was identified as DEG if |log2 fold change| ≥ 1 and *q* value ≤ 0.05. Moreover, the expression values for all identified DEGs across all the samples of roots and leaves is shown and clustered in Figures 3C,D.

### Gene Ontology Terms Enriched by Differentially Expressed Genes

**FIGURE 4:**
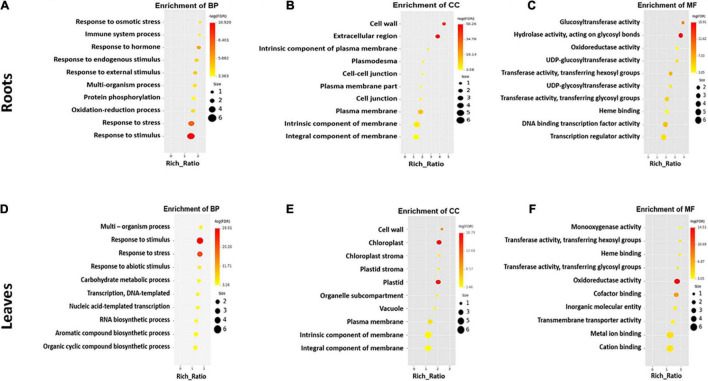
The most enriched GO terms in different categories viz., biological process **(A)**, cellular component **(B)**, and molecular function **(C)** in the roots, whereas biological process **(D)**, cellular component **(E)**, and molecular function **(F)** in the leaves of *S. argel* plants are exposed to salt stress.

The most enriched GO terms in the roots and leaves of *S. argel* plants are shown in Figure 4. Interestingly, “response to stress,” “response to osmotic stress,” “response to stimulus,” and “response to external and endogenous stimulus” were among the most enriched BP terms in roots (Figure 4A). “Transcription regulator” and “DNA binding transcription factor” activities were among the most enriched molecular function (MF) terms (Figure 4C). Furthermore, “UDP-glycotransferase” activity was one of the enriched MF terms in the roots of *S. argel* plants. The majority of enriched cellular component (CC) terms have relations with the cell membrane, e.g., “kintrinsic and integral components of membrane” and “plasma membrane” terms (Figure 4B).

Similarly, in the leaves of *S. argel* plants, the most enriched BP terms included “response to stimulus, stress, and abiotic stimulus” (Figure 4D). Interestingly, “biosynthesis of aromatic compounds and organic cyclic compounds” were also enriched in the leaves of *S. argel* plants that are exposed to salt stress. Similar to roots, CC terms with relation to “cellular membrane” were the most enriched under salt stress. Nevertheless, the terms related to “chloroplast and stroma” were enriched in the leaves (Figure 4E). However, the most enriched MF terms in the leaves of this plant included “monooxygenase activity” and “glycosyl and hexosyl transferase activity” (Figure 4F).

### Kyoto Encyclopedia of Genes and Genomes Pathways Enriched by Differentially Expressed Genes

**FIGURE 5:**
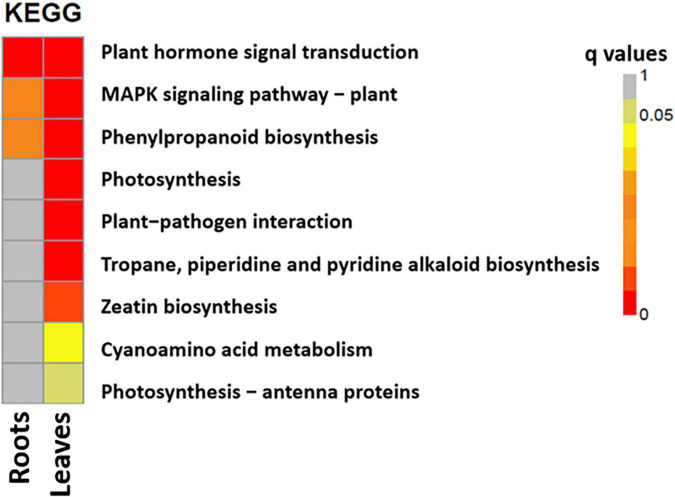
The most enriched KEGG pathways in the roots (T1_C1) and leaves (T2_C2) of *S. argel* plants are exposed to salt stress.

The most enriched KEGG pathways in the roots and leaves of *S. argel* plants under salt stress were identified. In addition, “plant hormone signal transduction,” “mitogen-activated protein kinase (MAPK) signaling pathway,” and “phenylpropanoid biosynthesis pathways” were the most enriched in both the roots and the leaves. Interestingly, photosynthesis-related pathways were enriched in the leaves of *S. argel* plants under salt stress. Furthermore, “zeatin biosynthesis” and “cyanoamino acid metabolism” pathways were enriched in the leaves of *S. argel* plants under salt stress conditions (Figure 5).

### Salt-Responsive Genes in *S. argel* Plants

In the current study, plant hormone signal transduction, MAPK signaling pathway, and phenylpropanoid biosynthesis pathways were highly enriched by up-regulated genes in the roots and leaves of *S. argel* plants indicating a potential response to salt stress conditions *via* these pathways. Table 2 shows DEGs selected in the roots of *S. argel* plants under salt stress conditions in these three pathways. It shows many genes whose expression was up- and down-regulated. Ten key genes participate in the plant hormone signal transduction pathway (six up-regulated genes and four down-regulated genes), two key genes participate in MAPK signaling pathway (one up-regulated gene and one down-regulated gene), and sixteen key genes (ten up-regulated genes and six down-regulated genes) participate in different metabolic pathways, such as amino acid metabolism, polyamine (PA) biosynthesis, phenylpropanoid biosynthesis, starch and sucrose metabolism which are possibly linked to salt tolerance in the roots and/or leaves of *S. argel* plants.

**TABLE 2:** Differentially expressed genes (DEGs) selected in the roots of *S. argel* plants under salt stress conditions.

NR_TopHit	Log2FC	pval	KO
**map04075| Plant hormone signal transduction**			
gi| 661887328	5.72	9.2E-12	K14497| PP2C; protein phosphatase 2C
gi| 731377363	1.29	1.1E-03	K14487| GH3; auxin responsive GH3 gene family
gi| 356556539	1.20	1.5E-03	K14484| IAA; auxin-responsive protein IAA
gi| 661899725	1.12	2.6E-09	K14488| SAUR; SAUR family protein
gi| 661883074	1.11	1.1E-03	K14491| ARR-B family response regulator
gi| 566187305	1.09	3.15E-06	K14432| ABF; ABA responsive element binding factor
gi| 661883380	–1.20	2.6E-04	K14496| PYL; abscisic acid receptor PYR/PYL family
gi| 802607484	–1.42	1.6E-03	K14488| SAUR; SAUR family protein
gi| 661882883	–1.76	1.3E-05	K13946| AUX1; auxin influx carrier
gi| 661882883	–2.37	1.8E-04	K14498| SNRK2; serine/threonine-protein kinase
**map04016| MAPK signaling pathway**			
gi| 308445435	2.60	4.3E-33	K20547| CHIB; basic endochitinase
gi| 747103681	–2.67	1.4E-04	K20718| ER; receptor-like serine/threonine-protein kinase
**map01100| Metabolic pathways**			
gi| 661890327	1.91	7.0E-08	K01188| beta-glucosidase
gi| 661892393	1.63	8.1E-06	K11816| YUCCA; indole-3-pyruvate monooxygenase
gi| 698455889	1.61	1.5E-13	K00430| peroxidase
gi| 675174201	22.43	2.8E-06	K12657| P5CS; delta-1-pyrroline-5-carboxylate syn.
gi| 85068614	3.71	1.9E-12	K05280| flavonoid 3′-monooxygenase
gi| 661895189	2.50	5.2E-05	K00826| ilvE; branched-chain aa aminotransferase
gi| 747094524	2.14	4.4E-04	K00827| AGXT2; alanine-glyoxylate transaminase
gi| 698534104	1.46	2.6E-09	K05909| laccase
gi| 747055899	0.70	7.7E-02	K01626| aroF; 3-deoxy-7-phosphoheptulonate syn.
gi| 661895601	0.08	8.7E-01	K08235| Xyloglucan: xyloglucosyl transferase
gi| 661880328	–0.24	8.3E-01	K00815| TAT; tyrosine aminotransferase
gi| 258549503	–0.80	1.4E-01	K00083| cinnamyl-alcohol dehydrogenase
gi| 565400707	–0.99	1.9E-02	K01580| GAD; glutamate decarboxylase
gi| 661878365	–1.07	3.6E-07	K01850| Chorismate mutase
gi| 661892399	–1.56	4.6E-06	K13832| DHQ-SDH; 3-dehydroquinate dehydratase
gi| 700211354	–3.01	1.6E-03	K01188| beta-glucosidase

Similarly, Table 3 shows DEGs-selected in the leaves of *S. argel* plants under salt stress conditions in these three pathways. It shows many genes whose expression was up-and down-regulated; Eleven key genes participate in the plant hormone signal transduction pathway (Nine up-regulated genes and two down-regulated genes), and six key genes participate in the MAPK signaling pathway (Four up-regulated genes and two down-regulated genes).

**TABLE 3:** Differentially expressed genes (DEGs) selected in the leaves of *S. argel* plants under salt stress conditions.

NR_TopHit	Log2FC	pval	KO
**map04075| Plant hormone signal transduction**			
gi| 747056090	8.51	2.3E-07	K14431| TGA; transcription factor TGA
gi| 661893229	3.54	1.7E-09	K14488| SAUR; SAUR family protein
gi| 661897476	3.12	5.7E-12	K14509| ETR, ERS; ethylene receptor
gi| 697167011	3.09	1.2E-07	K14497| PP2C; protein phosphatase 2C
gi| 661892418	2.98	1.6E-11	K14493| GID1; gibberellin receptor GID1
gi| 661885802	2.79	4.1E-15	K14515| EBF1_2; EIN3-binding F-box protein
gi| 590687104	2.50	5.1E-04	K14487| GH3; auxin responsive
gi| 661899331	1.62	8.6E-06	K13449| PR1; pathogenesis-related protein 1
gi| 661888977	1.35	4.2E-04	K14486| ARF; auxin response factor
gi| 661896253	–1.09	6.1E-04	K13464| JAZ; jasmonate ZIM domain- protein
gi| 46038191	–1.24	2.5E-06	K14517| ERF1; ethylene-responsive TF
**map04016| MAPK signaling pathway**			
gi| 698584006	8.05	4.5E-06	K13447| RBOH; respiratory burst oxidase
gi| 661897510	6.26	6.3E-05	K14516| ERF1; ethylene-responsive TF
gi| 661884155	2.88	4.8E-06	K20772| ACC; aminocyclopropane-1-carboxylate syn.
gi| 728843711	1.28	0.02503	K20536| MPK3; mitogen-activated protein kinase 3
gi| 661898687	–1.42	6.9E-05	K20604| MKK9; mitogen-activated protein kinase 9
gi| 302180065	–1.69	1.7E-06	K20547| CHIB; basic endochitinase B
**map01100| Metabolic pathways**			
gi| 661871855	4.01	2.72E-03	K05349| bglX; beta-glucosidase
gi| 747055899	3.79	4.96E-06	K01626| aroF; 3-deoxy-7-phosphoheptulonate syn.
gi| 661873721	3.09	1.15E-03	K01188| beta-glucosidase]
gi| 658055315	2.99	2.35E-04	K05350| bglB; beta-glucosidase
gi| 460405583	2.57	2.19E-06	K00815| TAT; tyrosine aminotransferase
gi| 661895601	2.31	3.33E-06	K00128| aldehyde dehydrogenase
gi| 747043299	2.17	1.05E-03	K01915| GLUL; glutamine synthetase
gi| 661880491	2.15	7.95E-07	K13832| aroDE; 3-dehydroquinate dehydratase
gi| 7798554	2.14	4.74E-07	K10775| PAL; phenylalanine ammonia-lyase
gi| 346990426	2.13	1.16E-08	K01904| 4CL; 4-coumarate–CoA ligase
gi| 661891443	2.02	1.02E-04	K01087| otsB; trehalose 6-phosphate phosphatase
gi| 661894501	1.93	1.07E-08	K00430| peroxidase
gi| 1351206	1.91	8.71E-04	K00487| CYP73A; *trans*-cinnamate 4-monooxygenase
gi| 641861046	1.81	4.22E-03	K14454| GOT1; aspartate aminotransferase
gi| 698464873	1.74	5.95E-05	K00827| AGXT2; alanine-glyoxylate transaminase
gi| 747043153	1.42	1.50E-03	K09753| CCR; cinnamoyl-CoA reductase
gi| 661895189	1.31	4.92E-07	K00826| ilvE; BCAA aminotransferase
gi| 661880262	1.30	1.97E-05	K17839| PAO; polyamine oxidase
gi| 661899712	1.13	8.68E-03	K01476| arg; arginase
gi| 8134570	0.63	4.78E-02	K00549| 5-methyltetrahydropteroyltriglutamate
gi| 731393006	0.04	9.10E-01	K03801| lipB; lipoyl(octanoyl) transferase
gi| 525314083	–0.95	7.19E-03	K00830| AGXT; alanine-glyoxylate transaminase
gi| 756786793	–1.25	7.16E-08	K00660| CHS; chalcone synthase
gi| 258549503	–1.39	1.57E-03	K00083| cinnamyl-alcohol dehydrogenase
gi| 848914114	–2.13	2.53E-04	K17055| EGS1; eugenol synthase

Twenty-five (21 up-regulated genes and four down-regulated genes) key genes participate in different metabolic pathways, such as amino acid metabolism, PA biosynthesis, phenylpropanoid biosynthesis, starch and sucrose metabolism, which are possibly linked to salt tolerance in the roots and/or leaves of *S. argel* plants.

### Plant Hormone Genes Enriched in *S. argel* Plants Under Salt Stress

**FIGURE 6:**
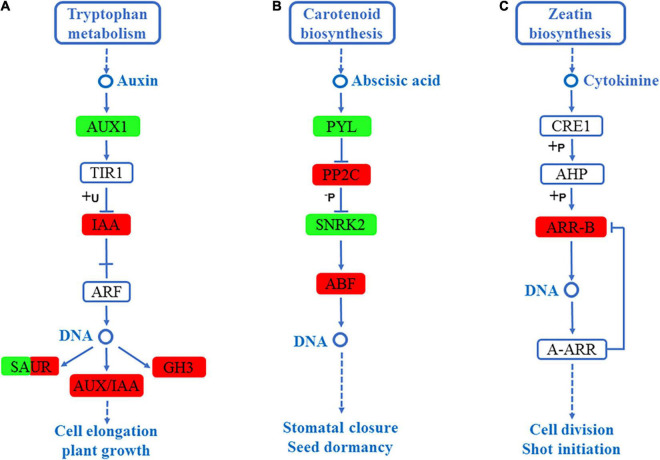
DEGs mapped to the plant hormone signaling transduction pathways in the roots of *S. argel* plants under salt stress conditions. **(A)** Auxin signaling pathway, **(B)** ABA signaling pathway, and **(C)** Cytokinin signaling pathway. The red box indicates up-regulated compared with the control. At the same time, the green box indicates down-regulated compared with the control: the fold change (FC) and *P*-value ≤ 0.05 are shown in Table 2.

The most enriched KEGG pathway in the roots and leaves of *S. argel* plants under salt stress was plant hormone signal transduction. In the roots, three hormone signal transduction pathways, including auxin signaling pathway, abscisic acid (ABA) signaling pathway, and cytokinin signaling pathway, were enriched. In the auxin signaling pathway, the expression level of auxin-responsive GH3 (*GH3*), auxin-responsive protein (*IAA*), and SAUR family protein (*SAUR*) were significantly high, whereas auxin influx carrier (*AUX1*) and the SAUR family protein (*SAUR)* had significantly lower expression level as shown in Figure 6A. In the ABA signaling pathway, the expression level of protein phosphatase 2C (*PP2C*) and ABA-responsive element-binding (*ABF*) were significantly up-regulated, whereas ABA receptor PYR/PYL (*PYL*) and serine/threonine-protein kinase (*SNRK2*) were significantly down-regulated as shown in Figure 6B. Finally, in the cytokinin signaling pathway, the ARR-B family response regulator (ARR-B) expression level was up-regulated, as shown in Figure 6C.

**FIGURE 7:**
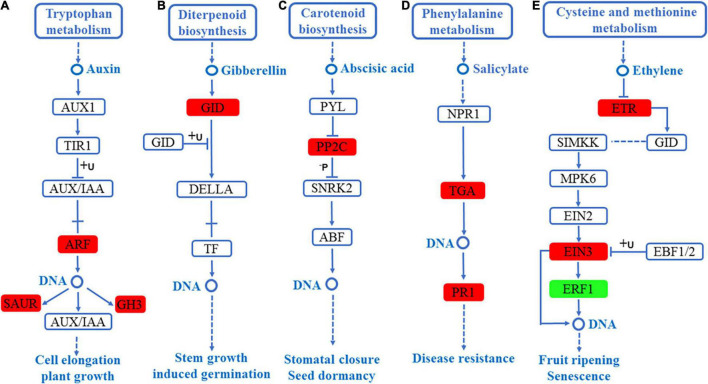
DEGs mapped to the plant hormone signaling transduction pathways in the leaves of *S. argel* plants under salt stress conditions. **(A)** Auxin signaling pathway, **(B)** gibberellin signaling pathway, **(C)** ABA signaling pathway, **(D)** salicylate signaling pathway, and **(E)** ethylene signaling pathway. The red box r indicates up-regulated pathway compared with the control. At the same time, the green box indicates down-regulated pathway compared with the control. The fold change (FC) and *P*-value ≤ 0.05 are shown in Table 3.

Similarly, in the leaves, five hormone signal transduction pathways, including auxin signaling pathway, gibberellin signaling pathway, ABA signaling pathway, salicylate signaling pathway, and ethylene signaling pathway, were enriched. In the auxin signaling pathway, the expression level of auxin-responsive *GH3*, *SAUR*, and auxin response factor (*ARF*) were significantly high, as shown in Figure 7A. In the gibberellin signaling pathway, the expression level of gibberellin receptor (*GID1*) was significantly up-regulated, as shown in Figure 7B. In the ABA signaling pathway, the expression level of PP2C was significantly high, as shown in Figure 7C. In the salicylate signaling pathway, the transcription factor (*TGA*) and pathogenesis-related protein 1 (*PR1*) were up-regulated, as shown in Figure 7D. Finally, in the ethylene signaling pathway, ERS ethylene receptor (*ETR*) and EIN3-binding F-box protein (*EIN3*) were up-regulated, whereas ethylene-responsive TF (*ERF1*) was significantly down-regulated, as shown in Figure 7E. Interestingly, the auxin-responsive *GH3* and protein phosphatase 2C *PP2C* were significantly up-regulated in the roots and leaves of *S. argel* plants (Figures 6A,B, 7A,C).

### Metabolome Profiling

**FIGURE 8:**
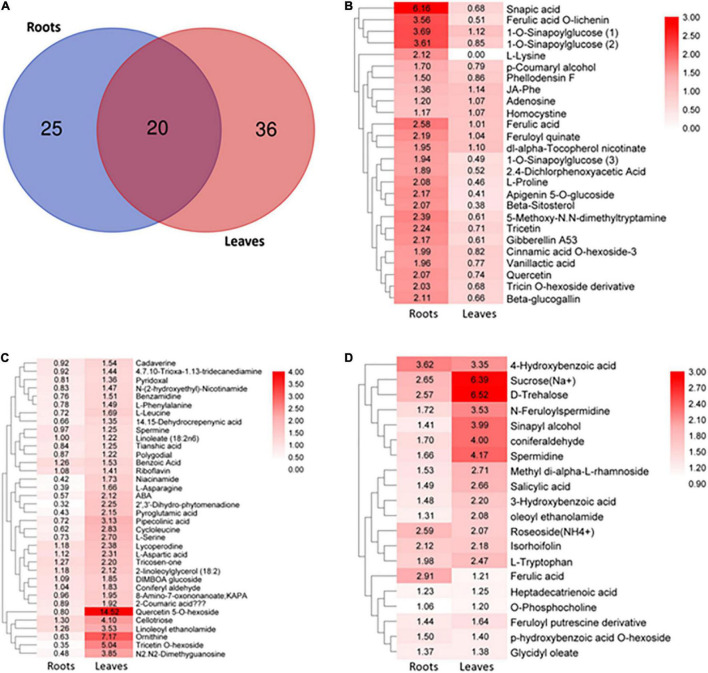
Overview of targeted metabolites in *S. argel* plants based on liquid chromatography-tandem mass spectrometry (LC-MS/MS) analysis. **(A)** Differentially accumulated metabolites in the stressed roots (T1_C1) and leaves (T2_C2) as compared to the control ones of *S. argel* plants; for plants grown below 500 mM NaCl salt, compared with control (free salt), **(B)** metabolites accumulated in the roots significantly while they accumulated insignificantly in the leaves, **(C)** metabolites accumulated significantly in the leaves while they accumulated insignificantly in the roots and **(D)** metabolites accumulated significantly both in the roots and leaves. The number in box plots reflects fold change and the bar on the right reflects log2 fold-change value of the accumulated metabolites. Replications (*n* = 6).

To examine the changes in the metabolism of *S. argel* plants under salt condition, the roots and the leaves were subjected to metabolomic assays using liquid chromatography-tandem mass spectrometry. Samples obtained from the roots under control or stressed conditions were compared together and those obtained from the leaves either under control or stressed conditions were also compared together. This analysis resulted in an estimated 233 known metabolites to minimize data uncertainty and help point out discrepancies between the roots and leaves; the metabolites were determined using an unpaired *t*-test (*P*-value < 0.05; fold change > 1). In stressed roots, 45 metabolites out of 87 metabolites were identified significant as compared to the control ones, while in the stressed leaves, 56 metabolites out of 112 metabolites were identified significant as compared to the control ones. There were 20 metabolites in common between the leaves and roots (Figure 8A).

Distinctive accumulative patterns of amino acids, phenylpropanoids, monolignols, flavonoids, saccharides, lipids, organic acids, and other compounds in the roots and leaves have been observed. Indeed, the levels of monolignols (sinapic acid, 1-o-sinapoylglucose, ferulic acid o-lichenin, cinnamic acid o-hexoside-3, and paracoumaryl alcohol accumulated), flavonoids (tricetin, apigenin 5-o-glucoside, quercetin, and tricin o-hexoside derivative), amino acids (L-proline, L-lysine, and homocystine), and organic acids (feruloyl quinate and vanillactic acid) were accumulated significantly in the roots but not in the leaves (Figure 8B).

In contrast, the levels of monolignols (tricosen-one, 2-coumaric acid, and coniferyl aldehyde), flavonoids (quercetin 5-o-hexoside and tricetin o-hexoside), amino acids (ornithine, l-serine, L-aspartic acid, L-leucine, L-asparagine, and L-phenylalanine), organic acids (pipecolinic acid, cycloleucine, pyroglutamic acid, and benzoic acid), PAs (cadaverine, benzamidine, and spermine), and other compounds (cellotriose and ABA) were significantly accumulated only in the leaves (Figure 8C). Interestingly, the levels of monolignols (ferulic acid, sinapyl alcohol, and coniferaldehyde), hydroxybenzoic acid derivatives (salicylic acid, p-hydroxybenzoic acid o-hexoside, 4-hydroxybenzoic acid, and 3-hydroxybenzoic acid), amino acids (L-tryptophan), phenolamines (n-feruloylspermidine and feruloyl putrescine derivative), PAs (spermidine), and saccharides (trehalose and sucrose) showed significant accumulation both in the roots and leaves (Figure 8D). These results indicate that many secondary metabolites derived from phenylalanine in the phenylpropanoid pathway were accumulated both in the roots and leaves of *S. argel* plants in response to salt stress. Moreover, some of the metabolites acting in ROS scavenging and osmotic adjustment (osmolytes) were accumulated, i.e., trehalose, sucrose, proline, and asparagine.

### qRT-PCR Validation

**FIGURE 9:**
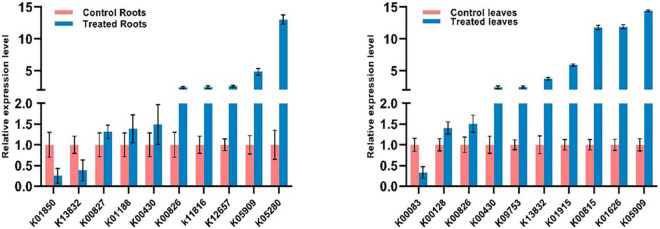
Changes in the expression of 10 selected DEGs in the roots (left) and 10 selected DEGs in the leaves (right) of *S. argel* plants in response to salt stress based on qRT-PCR results.

The expression levels of 20 different genes involved in the metabolic pathways were examined using qRT-PCR to validate their analysis using RNA-Seq data. The primers used for each gene are shown in Table 1. The expression levels of 20 genes (ten roots and ten leaves) by qRT-PCR in Figure 9 were consistent with RNA-Seq data. Consequently, the RNA-seq results were reliable for identifying and measuring the expression of DEGs involved in various processes in the *S. argel* plants in response to salt stress.

**FIGURE 10:**
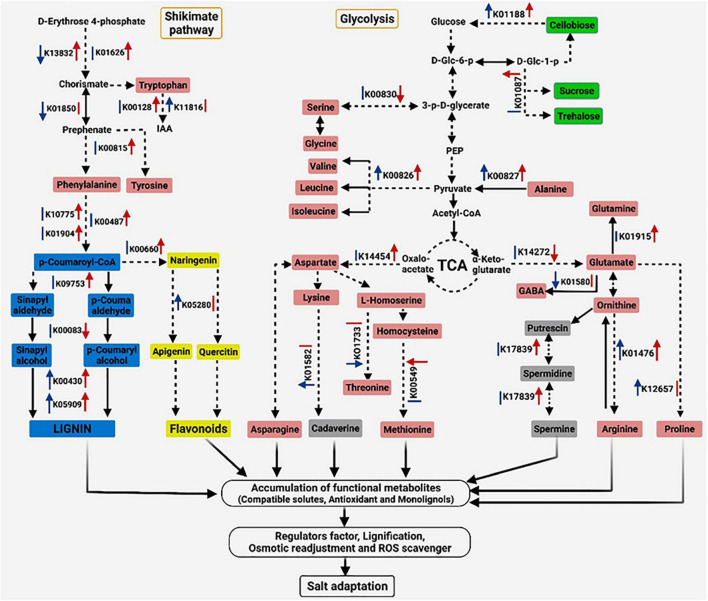
Proposed schematic of the adaptive mechanism in *S. argel* plants exposed to salt conditions. The red arrow on the right of each Kyoto Encyclopedia of Genes and Genomes (KEGG) number indicates a change in the gene expression level in the leaves and blue on the left indicates a change in the gene expression level in the roots; up arrows indicate upregulation while down arrows indicate downregulation with *q* value ≤ 0.05, except five genes (K00830, K01476, K01580, K01733, and K14454) with *P*-value ≤ 0.05, and the lines indicate no change. Different colors are used to show amino acids (pink), PAs (gray), flavonoids (yellow), phenylpropanoids (blue), disaccharides (green), and other metabolites (without color), i.e., IAA, Indole-3-acetic acid; D-Glc-6-p, D-Glucose 6-phosphate; D-Glc-1-p, D-Glucose 1-phosphate; 3-p-D-glycerate, 3-Phospho-D-glycerate; and PEP, Phosphoenolpyruvate.

Based on the previous presented results, a potential adaptive mechanism of salinity stress in *S*. *argel* was proposed and presented in Figure 10.

## Discussion

Salt stress is one of the most severe abiotic stresses affecting plant growth and productivity. Around 20% of the irrigated area and 6% of the overall land area of the world is sacrificed due to saline conditions (Shabala and Munns, 2017). From a physiological point of view, the limited plant growth and productivity caused by saline environment is due to salt stress on the chemical composition and activities *via* different impairing physiological aspects, including protein synthesis, lipid metabolism, photosynthesis, etc., (Shu et al., 2017). Therefore, the plants in arid and semi-arid regions that are exposed to high levels of various abiotic stresses, especially salt and drought might provide an ideal model to understand genes and metabolites that play critical roles in response to abiotic stress. Furthermore, concerning strategies to enhance crop tolerance, these plants may provide valuable insights into salt tolerance mechanisms (Gu J. et al., 2018; Benjamin et al., 2019). In the current study, we examined potential genes and metabolites that have pivotal roles in the salt adaptation of *S. argel* plants *via* examining transcriptomic and metabolomic changes in both the roots and leaves under salt stress.

Production of ROS is increased significantly in plant cells exposed to salinity stress. These species are highly toxic to macromolecules in the cells leading to secondary oxidative stress (Perrineau et al., 2014). Furthermore, ROS accumulation in the cells adversely affects the expression of many genes and impairs several vital processes including growth, programmed cell death, and signal transduction (Baxter et al., 2013). The excess Na^+^ and oxidative stress in the intra- or extra-cellular environment activate the cytoplasmic Ca^2+^ signal pathway to regulate an osmotic adjustment or homeostasis regulating salt stress responses (Yang et al., 2017). The ability of halophytes and glycophytes to sustain ROS homeostasis varies. Halophytes have robust antioxidant systems that contain both enzymatic and non-enzymatic components to control ROS amount in the cell. As a result, halophytes have higher ROS amounts than glycophytes. In addition, halophytes had faster H_2_O_2_ signaling kinetics than the glycophytes (Ellouzi et al., 2011; Bose et al., 2014). Our investigation shows that the most enriched GO terms in the biological process category responded to osmotic stress, oxidation-reduction process, oxidoreductase activity, and cation binding in the roots and/or leaves of *S. argel* plants were exposed to salt stress. Thus, we concluded that *S. argel* plants have a potential response mechanism when exposed to severe salt conditions. Combining the metabolomic results and transcriptomic data, much knowledge was gained about the metabolic pathways, such as amino acid metabolism, PA biosynthesis, phenylpropanoid biosynthesis, starch and sucrose, linked to salt tolerance in the roots and/or leaves of *S. argel* plants. In plants, free amino acids can be used in osmotic adjustment, scavenging of ROS, stabilization of proteins, membranes, and subcellular structures to control salt-induced osmotic stress. In this study, the physiological levels of proline, lysine, and homocysteine increased significantly in the roots: ornithine, serine, aspartic acid, leucine, and asparagine L-phenylalanine increased significantly in the leaves while tryptophan increased significantly in the roots and leaves of *S. argel* plants. Thus, proline is a multifunctional amino acid in the plant defense system and is a charged metabolite to osmotic adjustment and ROS scavenger (Szabados and Savouré, 2010; Yang and Guo, 2018). Plant hormones (phytohormones) control the growth of plants. The ABA, ethylene, jasmonic acid (JA), and salicylic acid (SA) are classified as stress-reactive hormones. At the same time, auxin is known as a growth and development promoting hormone (Verma et al., 2016; Yu et al., 2020). ABA accumulation is a notable factor in water stress induced by a hyperosmotic signal that causes several adaptive reactions in plants (Zhu, 2002). In the current study, several functional genes participating in stress reaction hormones, such as ABA signaling pathway, salicylate signaling pathway, and ethylene signaling pathway were up-regulated in treated samples compared to the controls. For example, the PP2C gene was up-regulated in the roots and leaves by 5.72 and 3.09-fold, respectively, whereas ABF was up-regulated only in the roots (Figure 6B). PP2Cs bind to SnRK2 kinases (OST1) in the absence of ABA to keep the kinases inactive by removing the phosphorylation of activation loop (Soon et al., 2014). Moreover, several clades, such as PP2C protein phosphatases interact directly with RopGEF1 to create a RopGEF-ROP-PP2C control loop model that is thought to help shut down ABA signal transduction. In *Arabidopsis*, *AtPP2CG1* positively regulates salt tolerance and is expressed in the vascular trichomes (Liu et al., 2012). Similarly, *OsPP108* (a group A PP2C) confers salt stress tolerance in *Arabidopsis* via negatively regulate ABA signaling (Singh et al., 2015). Moreover, in *Betula platyphylla*, *BpPP2C1* was highly expressed and in roots, the *BpPP2C1* possibly confers salt tolerance by enhancing the metabolic activity of flavanols, anion transport, and oxidative stress. Together, these results confirm that PP2C family members are involved in the regulation of abiotic stress (Xing et al., 2021). Also, ABF2 was involved in glucose signaling and salt stress tolerance in *Arabidopsis* (Kim et al., 2004). Together, our results showed that *PP2C* was highly expressed in the roots and leaves of *S. argel* plants, whereas *ABF* was highly expressed only in the roots, indicating that *PP2C* and *ABF* may involve in the response of *S. argel* plants to saline stress. These observations indicate that these genes possibly play a role in the response of *S*. *argel* plants to salt stress.

In *Arabidopsis*, the salicylic acid signaling pathway is important for improved osmotic stress tolerance (Jayakannan et al., 2015). In *S. argel* plants, *TGA* and *PR1* were highly expressed in the leaves by 8.51 and 1.62-fold, respectively; these observations, taken together, suggest that *TGA* and *PR1* genes have participated in the response mechanism to salt stress in *S. argel* plants.

It has been demonstrated that auxin plays a key role in mediating plant growth and development under stress conditions, such as salt. Exogenous application with auxin, such as IAA resulted in increased salt stress tolerance in several crops (Ribba et al., 2020). Endogenous maintenance of auxin distribution and the particular auxin pattern in the roots are required for the normal growth and development. Auxin concentration is affected by local auxin biosynthesis and auxin transporters. YUCCA is the most important and widely investigated pathway for local auxin biosynthesis (Zhao, 2018). Our results showed that under salt stress, the expression level of *YUCCA* gene was increased in the roots by 1.63-fold; also, auxin-responsive GH3 (*GH3*) was up-regulated in the roots and leaves of *S. argel* plants by 1.29 and 2.5-fold, respectively. Auxin-amido synthetases encoded by Gretchen Hagen 3 (GH3) protein family can regulate active auxin levels by conjugation with amino acids, indicating increased auxin biosynthesis of *YUCCA*, accompanied with inactivating auxin by *GH3*. Moreover, auxin influx (*AUX1*) transporter was down-regulated under this experimental condition. It was reported that *AUX1* is also important for proper plant growth and development and has been implicated in salt stress responses (Fàbregas et al., 2015). Taken together, our results revealed that salt stress significantly affected auxin homeostasis in *S. argel* plants by changing the local auxin biosynthesis, auxin influx carriers, and the level of active auxin in the cell. This pathway could be part of a mechanism that *S. argel* plants use to maintain the salt stress.

Proline is known for its central role in salinity tolerance in plants. Furthermore, the literature also suggests that pyrroline-5-carboxylate synthetase (P5CS) is a central enzyme in proline synthesis in plants. In our study, proline accumulation and enzyme activity of P5CS (K12657) were increased only in the roots; this result was consistent with that was found in *S. maritima*, where proline accumulated only in the roots (Benjamin et al., 2019). Furthermore, the proline accumulation was associated with higher P5CS activity during drought stress in tobacco (Szepesi and Szőllősi, 2018). *P5CS* expression level increased in the root tip, shoot apex, leaves, and inflorescences under stress (Székely et al., 2008; Kavi Kishor and Sreenivasulu, 2014) but in our study, it was increased significantly only in the roots, which indicates that the relationship between P5CS expression level and proline accumulation is positive and tissue-specific.

Further, there is an evidence that asparagine often accumulates significantly at the same time as proline in drought and salt stress in various plants, e.g., soybean (*Glycine max*), alfalfa (*Medicago sativa*), pearl millet (*Cenchrus americanus*), and wheat (*Triticum aestivum*; Lea et al., 2007). Moreover, in the younger leaves, asparagine accumulated, while proline showed the reverse of this trend. The accumulation of asparagine only in the leaves, observed in our study, is consistent with the results obtained in barley plants where asparagine was leaf-specific solutes under salt stress (Wu et al., 2013). On the other hand, aspartic acid and aspartate aminotransferase (GOT1; K14454) enzyme increased only in the leaves. Previews of literature indicate that GOT1 catalyzes aspartic acid synthesis from glutamic acid or oxaloacetate in all organisms (de la Torre et al., 2014). Asparagine and aspartic acid responded similarly under salt stress in barley and soybean (Wu et al., 2013; Zhang et al., 2016). It could be attributed that asparagine results from aspartic acid (reverse reaction) metabolism. Our results indicate that both asparagine and aspartic acid may be involved in similar response mechanism operated under salt stress.

Interestingly, ornithine (Orn) increased by 7.17-fold in the stressed leaves as compared to the control ones. Besides, the expression level of arginase (*ARG*; K01476) was increased both in the roots and leaves. Indeed, Orn biosynthesis pathway depends upon stress levels; a correlation has been observed between proline accumulation in *Arabidopsis* and *Brassica napus* plants and the ornithine pathway under prolonged conditions of severe stress (Kalamaki, 2009; Xue et al., 2009). It was found that the activation of *ARG* promotes Orn biosynthesis from Arg in *A. thaliana* plants exposed to bacterial infection (Jones et al., 2006). Hence, the metabolic pathways of pro, arginine, and Orn showed that Orn occupies a vital role in the three pathways (Anwar et al., 2018). Moreover, Orn contributes to PA metabolism.

All living organisms include PAs, which are small polycationic chemicals. Their unique chemical characteristics, such as low molecular weight, having two or more amino groups in their chemical structure, and positively charged compounds, give them a robust biological activity (Takahashi and Kakehi, 2009). Furthermore, PAs can influence the activity of DNA, RNA, proteins, and phospholipids (negatively charged molecules) by interacting with them (Igarashi and Kashiwagi, 2010).

Putrescine (Put), spermidine (Spd), spermine (Spm), and thermospermine (Tspm) are the most common PAs found in plants. There are accumulating evidences indicating that endogenous modification of PAs by exogenous applications of PAs, mainly Put, Spd, and Spm, protect against the effects of several forms of abiotic stress (Del Duca et al., 2014; Sequera-Mutiozabal et al., 2016). It has been demonstrated that increasing PA levels are one of the most notable metabolites in plants exposed to salt and other stresses. Alterations mainly produce the alteration in the levels of PAs in PA biosynthesis genes induced by stresses. Our study found that the expression level of Spd was increased by 4.17 and 1.66-fold in the leaves and roots, respectively, whereas Spm increased in the leaves by 1.25-fold while no change in Spm was observed in the roots. Polyamine balance is regulated by biosynthesis, back conversion, and terminal catabolism. Polyamine oxidases (PAOs) is involved in higher polyamine back conversion and terminal catabolism. Our study also found salt stress-induced *PAO2* expression, which may also be involved in increasing Spd and Spm by reverse biosynthesis process. However, the PA biosynthesis regulation is not well investigated. These findings indicate that Spd and Spm play a vital role in adaptation under salt stress conditions.

These findings were also found in wheat, where Spd and Spm significantly promoted grain filling and osmotic resistance (Liu et al., 2016). Additionally, the increased Spd and Spm by 3–4 times higher in transgenic rice lines improved the growth of the seedlings compared to non-transgenic lines under NaCl stress (Roy and Wu, 2002). Overall, our results indicate that the amount of Orn amino acid was tissue-specific and linked to salt levels. Further, Orn occupied a vital role in salt stress response, possibly by adjusting the biosynthesis levels of amino acids and PAs.

In a distinct pathway from that of the other well-characterized ornithine- or arginine-derived PAs, cadaverine (PA) comes from lysine. Cadaverine modulates the growth and responses to environmental stress (Jancewicz et al., 2016). Lysine decarboxylase (LDC; K01582) is mainly decarboxylated and transforms lysine into cadaverine. Moreover, quinolizidine alkaloids (QAs) are derived from cadaverine by the action of LDC (Bunsupa et al., 2012). In our study, the LDC was up-regulated in the roots while cadaverine was increased in the leaves. We suggest that this pathway may play a role in the response of *S. argel* plants to salt stress. However, it is interesting to explore cadaverine biosynthesis pathway under salt stress in further research.

A significant class of plant secondary metabolites is biosynthesized *via* the phenylpropanoids pathway. This pathway starts with amino acids (phenylalanine or/and tyrosine) which converts *via* phenylalanine ammonia-lyase (PAL; K10775), C4H (cinnamate 4-hydroxylase; K00487), and 4CL (4-coumaroyl CoA ligase; K01904) into p-coumaroyl. The latter acts as a core for the phenylpropanoid pathway. At the end of this very complex metabolic pathway, all flavonoids, lignins, and several intermediate molecules are synthesized, whose exact biological roles correlate with the stress management strategies of plants (Zhang et al., 2015; Le Roy et al., 2016). Flavonoids in different classes, such as flavonoids (e.g., naringenin), isoflavone (e.g., isorhoifolin), and flavonols (e.g., quercetin) have vital roles during abiotic stresses to plant protection. Interestingly, the backbone of flavonoid is synthesized by chalcone synthase (CHS; K00660), which generates particular flavonoids regulated by diverse enzymes, such as F3′H (flavonoid 3-hydoxylase; K05280; Gu F. et al., 2018; Pi et al., 2019). Our data showed that the salt stress up-regulated the expression of phenylpropanoids biosynthesis-related genes, such as *PAL*, *C4H*, *4CL*, and *CHS* in the leaves while *F3H* was up-regulated in the root tissues. Simultaneously, both the flavonoids, quercetin 5-O-hexoside and tricetin were increased by 14.52 and 5.04-fold in the stressed leaves. Meanwhile, quercetin and tricetin were increased by 2.07 and 2.24-fold in the roots, respectively. Interestingly, isorhoifolin was increased by 2.12 and 2.18-fold in the stressed roots and leaves, respectively, indicating that these flavonoids probably play critical roles in the responses of *S. argel* plants to salt stress.

Notably, hydroxycinnamic acids (HCAAs) derived from the phenylpropanoid pathway can combine with the PAs, which are referred to as polyamine conjugates or HCAAs, e.g., hydroxyferuloyl and hydroxycinnamoyl spermidine conjugates. The HCAAs are involved in various growth and development processes and responses to environmental stress in plants (Luo et al., 2009). Thus, the increase of N-feruloylspermidine and feruloyl putrescine derivatives in the roots and leaves indicates that these metabolites may play a role in the response of the *S. argel* plants to salt stress. It is likely to work to reverse the oxidative status imbalance caused by salt. Further, lignin accumulation is regulated by diverse enzymes in the following two steps: (i) cinnamoyl-CoA reductase (CCR; K09753) and cinnamyl-alcohol dehydrogenase (CAD; K00083) conversion of HCAAs derivatives to hydroxycinnamyl alcohols (Monolignols) and (ii) peroxidase (POD; K00430) and laccase (K05909) for polymerizing monolignols to lignin polymer to support cell wall formation (Wang et al., 2014). Changes in the biosynthesis of lignin affects plant growth and protection (Xie et al., 2018). In the *S. argel* plants, both peroxidase and laccase were up-regulated in the stressed roots and leaves. Coinciding with this increase in gene expression, several HCAAs were also increased (Figure 8D).

The key players in different metabolic and regulatory processes are amino acids and carbohydrates. During the response to salt stress in tomatoes, 17 amino acids and 17 carbohydrate metabolic pathways were found enriched (Thalmann and Santelia, 2017; Zhang et al., 2017). Salt tolerance significantly affected the core genes related to starch and sucrose metabolism of *Arachis hypogaea* (Zhang H. et al., 2020). In general, salt stress significantly impacted the increased accumulation of trehalose and sucrose in the roots and leaves of *S. argel* plant (Figure 8D) and increased the gene expression of beta-glucosidase (*BGL*; K01188). *BGL* was increased in the roots and shoots of maize following salt treatment (Zörb et al., 2004). Our data indicate that trehalose and sucrose may play a pivotal role in response to severe salt stress. According to the findings of other studies, trehalose and sucrose act as compatible osmolytes, minimize short-term water loss, and enhance cell turgor and cell expansion under long-term osmotic stress (Ruan, 2014; Henry et al., 2015).

## Conclusion

Profiling of transcriptomic and metabolimoc changes happened during exposure to different abiotic stresses may provide a deep insight into the potential mechanisms behind stress tolerance in plants. In the current study, integrating transcriptomics and metabolomic changes revealed that several genes and metabolomic pathways might play pivotal roles in the salinity stress of *S*. *argel* plants. The results of the current study provide a comprehensive database for the transctomic and metabolomic profiles and the potential changes happened at these levels under salinity stress in *S*. *argel* plants. Furthermore, the obtained results in the current study lay the foundation for the future biotechnological studies aiming to examine the potential salinity tolerance mechanisms in *S*. *argel* plants and might help in the etabolic engineering efforts aiming to enhance the salinity stress in sensitive plants. Nevertheless, further comprehensive analyses of the pathways and mechanisms identified and proposed in the current study are needed either in the same plant or in different salt-tolerant plants.

## Data Availability Statement

The datasets presented in this study can be found in NCBI-SRA database under BioSample accessions numbers SAMN21419729 and SAMN21419730.

## Author Contributions

HA: conceptualization, software, formal analysis, writing – original draft, and visualization. MM: software, formal analysis, data curation, and investigation. EA-S: software, writing original draft, and visualization. YL: software and validation. CY: methodology and formal analysis. NE: resources and investigation. ME: investigation and validation. EN: conceptualization and writing–review and editing. JL: supervision, project administration, and funding acquisition. All authors contributed to the article and approved the submitted version.

## Conflict of Interest

The authors declare that the research was conducted in the absence of any commercial or financial relationships that could be construed as a potential conflict of interest.

## Publisher’s Note

All claims expressed in this article are solely those of the authors and do not necessarily represent those of their affiliated organizations, or those of the publisher, the editors and the reviewers. Any product that may be evaluated in this article, or claim that may be made by its manufacturer, is not guaranteed or endorsed by the publisher.

## Footnotes

1
http://www.kegg.jp/

## Supplementary Material

Supplementary Figure 1 Salinity influence on phenotypic characteristics of *S*. *argel* seedlings after 3-days treatment in a greenhouse. Control: control seedlings, treated: seedlings treated with 500 mM NaCl for 3 days.

## References

[B1] Al-Juhaimi F. Y. Shahzad S. A. Ahmed A. S. Adiamo O. Q. Mohamed Ahmed I. A. Alsawmahi O. N. (2018). Effect of argel (*Solenostemma argel*) leaf extract on quality attributes of chicken meatballs during cold storage. *J. Food Sci. Technol.* 55 1797–1805. 10.1007/s13197-018-3094-1 29666532 PMC5897300

[B2] Anders S. Pyl P. T. Huber W. (2015). HTSeq-a Python framework to work with high-throughput sequencing data. *Bioinformatics* 31 166–169. 10.1093/bioinformatics/btu638 25260700 PMC4287950

[B3] Anwar A. She M. Wang K. Riaz B. Ye X. (2018). Biological roles of ornithine aminotransferase (OAT) in plant stress tolerance: present progress and future perspectives. *Int. J. Mol. Sci.* 19 3681–3700. 10.3390/ijms19113681 30469329 PMC6274847

[B4] Baxter A. Mittler R. Suzuki N. (2013). ROS as key players in plant stress signalling. *J. Exp. Bot.* 65 1229–1240. 10.1093/jxb/ert375 24253197

[B5] Benjamin J. J. Lucini L. Jothiramshekar S. Parida A. (2019). Metabolomic insights into the mechanisms underlying tolerance to salinity in different halophytes. *Plant Physiol. Biochem.* 135 528–545. 10.1016/j.plaphy.2018.11.006 30442441

[B6] Bose J. Rodrigo-Moreno A. Shabala S. (2014). ROS homeostasis in halophytes in the context of salinity stress tolerance. *J. Exp. Bot.* 65 1241–1257. 10.1093/jxb/ert430 24368505

[B7] Bryant D. M. Johnson K. Ditommaso T. Tickle T. Couger M. B. Payzin-Dogru D. (2017). A tissue-mapped axolotl *de novo* transcriptome enables identification of limb regeneration factors. *Cell Rep.* 18 762–776. 28099853 10.1016/j.celrep.2016.12.063 PMC5419050

[B8] Bunsupa S. Katayama K. Ikeura E. Oikawa A. Toyooka K. Saito K. (2012). Lysine decarboxylase catalyzes the first step of quinolizidine alkaloid biosynthesis and coevolved with alkaloid production in leguminosae. *Plant Cell* 24 1202–1216. 10.1105/tpc.112.095885 22415272 PMC3336119

[B9] Chen W. Gao Y. Xie W. Gong L. Lu K. Wang W. (2014). Genome-wide association analyses provide genetic and biochemical insights into natural variation in rice metabolism. *Nat. Genet.* 46 714–721. 10.1038/ng.3007 24908251

[B10] Chen W. Gong L. Guo Z. Wang W. Zhang H. Liu X. (2013). Integrated method for large-scale detection, identification, and quantification of widely targeted metabolites: application in the study of rice metabolomics. *Mol. Plant* 6 1769–1780. 10.1093/mp/sst080 23702596

[B11] Conesa A. Gotz S. Garcia-Gomez J. M. Terol J. Talon M. Robles M. (2005). Blast2GO: a universal tool for annotation, visualization and analysis in functional genomics research. *Bioinformatics* 21 3674–3676. 10.1093/bioinformatics/bti610 16081474

[B12] de la Torre F. El-Azaz J. Ávila C. Cánovas F. M. (2014). Deciphering the role of aspartate and prephenate aminotransferase activities in plastid nitrogen metabolism. *Plant Physiol.* 164 92–104. 10.1104/pp.113.232462 24296073 PMC3875828

[B13] Del Duca S. Serafini-Fracassini D. Cai G. (2014). Senescence and programmed cell death in plants: polyamine action mediated by transglutaminase. *Front. Plant Sci.* 5:120. 10.3389/fpls.2014.00120 24778637 PMC3985020

[B14] Dresen S. Ferreirós N. Gnann H. Zimmermann R. Weinmann W. (2010). Detection and identification of 700 drugs by multi-target screening with a 3200 Q TRAP^®^ LC-MS/MS system and library searching. *Anal. Bioanal. Chem.* 396 2425–2434. 10.1007/s00216-010-3485-2 20127316

[B15] El-Shiekh R. A. Salem M. A. Mouneir S. M. Hassan A. Abdel-Sattar E. (2021). A mechanistic study of *Solenostemma argel* as anti-rheumatic agent in relation to its metabolite profile using UPLC/HRMS. *J. Ethnopharmacol.* 265:113341. 10.1016/j.jep.2020.113341 32891814

[B16] Ellouzi H. Ben Hamed K. Cela J. Munné-Bosch S. Abdelly C. (2011). Early effects of salt stress on the physiological and oxidative status of *Cakile maritima* (halophyte) and *Arabidopsis thaliana* (glycophyte). *Physiol. Plant.* 142 128–143. 10.1111/j.1399-3054.2011.01450.x 21288246

[B17] Fàbregas N. Formosa-Jordan P. Confraria A. Siligato R. Alonso J. M. Swarup R. (2015). Auxin influx carriers control vascular patterning and xylem differentiation in *Arabidopsis thaliana*. *PLoS Genet.* 11:e1005183. 10.1371/journal.pgen.1005183 25922946 PMC4414528

[B18] Gan T. Lin Z. Bao L. Hui T. Cui X. Huang Y. (2021). Comparative proteomic analysis of tolerant and sensitive varieties reveals that phenylpropanoid biosynthesis contributes to salt tolerance in mulberry. *Int. J. Mol. Sci.* 22:9402. 10.3390/ijms22179402 34502318 PMC8431035

[B19] Grabherr M. G. Haas B. J. Yassour M. Levin J. Z. Thompson D. A. Amit I. (2011). Full-length transcriptome assembly from RNA-Seq data without a reference genome. *Nat. Biotechnol.* 29 644–652. 10.1038/nbt.1883 21572440 PMC3571712

[B20] Gu F. Wu G. Fang Y. Zhu H. (2018). Nontargeted metabolomics for phenolic and polyhydroxy compounds profile of Pepper (*Piper nigrum* L.) products based on LC-MS/MS analysis. *Molecules* 23 1985–1999. 10.3390/molecules23081985 30096911 PMC6222777

[B21] Gu J. Xia Z. Luo Y. Jiang X. Qian B. Xie H. (2018). Spliceosomal protein U1A is involved in alternative splicing and salt stress tolerance in *Arabidopsis thaliana*. *Nucleic Acids Res.* 46 1777–1792. 10.1093/nar/gkx1229 29228330 PMC5829640

[B22] Guan L. Haider M. S. Khan N. Nasim M. Jiu S. Fiaz M. (2018). Transcriptome sequence analysis elaborates a complex defensive mechanism of grapevine (*Vitis vinifera* l.) in response to salt stress. *Int. J. Mol. Sci.* 19:4019. 10.3390/ijms19124019 30545146 PMC6321183

[B23] Haider M. S. Kurjogi M. M. Khalil-Ur-Rehman M. Fiaz M. Pervaiz T. Jiu S. (2017). Grapevine immune signaling network in response to drought stress as revealed by transcriptomic analysis. *Plant Physiol. Biochem.* 121 187–195. 10.1016/j.plaphy.2017.10.026 29127881

[B24] Henry C. Bledsoe S. W. Griffiths C. A. Kollman A. Paul M. J. Sakr S. (2015). Differential role for trehalose metabolism in salt-stressed maize. *Plant Physiol.* 169 1072–1089. 10.1104/pp.15.00729 26269545 PMC4587459

[B25] Igarashi K. Kashiwagi K. (2010). Modulation of cellular function by polyamines. *Int. J. Biochem. Cell Biol.* 42 39–51. 10.1016/j.biocel.2009.07.009 19643201

[B26] Isayenkov S. V. Maathuis F. J. M. (2019). Plant salinity stress: many unanswered questions remain. *Front. Plant Sci.* 10:80. 10.3389/fpls.2019.00080 30828339 PMC6384275

[B27] Jancewicz A. L. Gibbs N. M. Masson P. H. (2016). Cadaverine’s functional role in plant development and environmental response. *Front. Plant Sci.* 7:870. 10.3389/fpls.2016.00870 27446107 PMC4914950

[B28] Jayakannan M. Bose J. Babourina O. Shabala S. Massart A. Poschenrieder C. (2015). The NPR1-dependent salicylic acid signalling pathway is pivotal for enhanced salt and oxidative stress tolerance in *Arabidopsis*. *J. Exp. Bot.* 66 1865–1875. 10.1093/jxb/eru528 25614660 PMC4378626

[B29] Jones A. M. E. Thomas V. Bennett M. H. Mansfield J. Grant M. (2006). Modifications to the *Arabidopsis* defense proteome occur prior to significant transcriptional change in response to inoculation with *Pseudomonas syringae*. *Plant Physiol.* 142 1603–1620. 10.1104/pp.106.086231 17028151 PMC1676056

[B30] Kalamaki M. S. (2009). Can ornithine accumulation modulate abiotic stress tolerance in *Arabidopsis*? *Plant Signal. Behav.* 4 1099–1101. 10.4161/psb.4.11.9873 19901538 PMC2819526

[B31] Kavi Kishor P. B. Sreenivasulu N. (2014). Is proline accumulation per se correlated with stress tolerance or is proline homeostasis a more critical issue? *Plant Cell Environ.* 37 300–311. 10.1111/pce.12157 23790054

[B32] Kim S. Kang J.-Y. Cho D.-I. Park J. H. Kim S. Y. (2004). ABF2, an ABRE-binding bZIP factor, is an essential component of glucose signaling and its overexpression affects multiple stress tolerance. *Plant J.* 40 75–87. 10.1111/j.1365-313X.2004.02192.x 15361142

[B33] Langmead B. Salzberg S. L. (2012). Fast gapped-read alignment with Bowtie 2. *Nat. Methods* 9 357–359. 10.1038/nmeth.1923 22388286 PMC3322381

[B34] Le Roy J. Huss B. Creach A. Hawkins S. Neutelings G. (2016). Glycosylation is a major regulator of phenylpropanoid availability and biological activity in plants. *Front. Plant Sci.* 7:735. 10.3389/fpls.2016.00735 27303427 PMC4880792

[B35] Lea P. J. Sodek L. Parry M. A. J. Shewry P. R. Halford N. G. (2007). Asparagine in plants. *Ann. Appl. Biol.* 150 1–26.

[B36] Liu X. Zhu Y. Zhai H. Cai H. Ji W. Luo X. (2012). *AtPP2CG1*, a protein phosphatase 2C, positively regulates salt tolerance of *Arabidopsis* in abscisic acid-dependent manner. *Biochem. Biophys. Res. Commun.* 422 710–715. 10.1016/j.bbrc.2012.05.064 22627139

[B37] Liu Y. Liang H. Lv X. Liu D. Wen X. Liao Y. (2016). Effect of polyamines on the grain filling of wheat under drought stress. *Plant Physiol. Biochem.* 100 113–129. 10.1016/j.plaphy.2016.01.003 26812255

[B38] Love M. I. Huber W. Anders S. (2014). Moderated estimation of fold change and dispersion for RNA-seq data with DESeq2. *Genome Biol.* 15:550. 10.1186/s13059-014-0550-8 25516281 PMC4302049

[B39] Luo J. (2015). Metabolite-based genome-wide association studies in plants. *Curr. Opin. Plant Biol.* 24 31–38. 10.1016/j.pbi.2015.01.006 25637954

[B40] Luo J. Fuell C. Parr A. Hill L. Bailey P. Elliott K. (2009). A novel polyamine acyltransferase responsible for the accumulation of spermidine conjugates in *Arabidopsis* seed. *Plant Cell* 21 318–333. 10.1105/tpc.108.063511 19168716 PMC2648071

[B41] Maher M. Ahmad H. Nishawy E. Li Y. Luo J. (2021). Novel transcriptome study and detection of metabolic variations in UV-B-treated date palm (Phoenix dactylifera cv. Khalas). *Int. J. Mol. Sci.* 22:2564. 10.3390/ijms22052564 33806362 PMC7961990

[B42] Mercado F. G. Torres F. D. M. Luque E. G. De Haro Lozano S. (2012). Salinity tolerance of the hygrophilous plant species in the wetlands of the south of the Iberian Peninsula. *Not. Bot. Horti Agrobot. Cluj Napoca* 40 18–28. 10.15835/nbha4017784

[B43] Mortazavi A. Williams B. A. Mccue K. Schaeffer L. Wold B. (2008). Mapping and quantifying mammalian transcriptomes by RNA-Seq. *Nat. Methods* 5 621–628. 10.1038/nmeth.1226 18516045 PMC13303166

[B44] Okur B. Örçen N. (2020). “Soil salinization and climate change,” in *Climate Change and Soil Interactions*, eds Prasad M. N. V. Pietrzykowski M. (Amsterdam: Elsevier), 331–350. 10.1016/B978-0-12-818032-7.00012-6

[B45] Ounaissia K. Pertuit D. Mitaine-Offer A. C. Miyamoto T. Tanaka C. Delemasure S. (2016). New pregnane and phenolic glycosides from *Solenostemma argel*. *Fitoterapia* 114 98–104. 10.1016/j.fitote.2016.08.002 27511059

[B46] Perrineau M. M. Zelzion E. Gross J. Price D. C. Boyd J. Bhattacharya D. (2014). Evolution of salt tolerance in a laboratory reared population of *Chlamydomonas reinhardtii*. *Environ. Microbiol.* 16 1755–1766. 10.1111/1462-2920.12372 24373049

[B47] Pi E. Xu J. Li H. Fan W. Zhu C. Zhang T. (2019). Enhanced salt tolerance of rhizobia-inoculated soybean correlates with decreased phosphorylation of the transcription factor *GmMYB183* and altered flavonoid biosynthesis. *Mol. Cell. Proteom.* 18 2225–2243. 10.1074/mcp.RA119.001704 31467032 PMC6823849

[B48] Plaza A. Perrone A. Balestrieri M. L. Felice F. Balestrieri C. Hamed A. I. (2005). New unusual pregnane glycosides with antiproliferative activity from *Solenostemma argel*. *Steroids* 70 594–603. 10.1016/j.steroids.2005.02.019 15946718

[B49] Ribba T. Garrido-Vargas F. O’brien J. A. (2020). Auxin-mediated responses under salt stress: from developmental regulation to biotechnological applications. *J. Exp. Bot.* 71 3843–3853. 10.1093/jxb/eraa241 32433743

[B50] Rossi L. Borghi M. Francini A. Lin X. Xie D. Y. Sebastiani L. (2016). Salt stress induces differential regulation of the phenylpropanoid pathway in *Olea europaea* cultivars Frantoio (salt-tolerant) and Leccino (salt-sensitive). *J. Plant Physiol.* 204 8–15. 10.1016/j.jplph.2016.07.014 27497740

[B51] Roy M. Wu R. (2002). Overexpression of S-adenosylmethionine decarboxylase gene in rice increases polyamine level and enhances sodium chloride-stress tolerance. *Plant Sci.* 163 987–992. 10.1016/S0168-9452(02)00272-8

[B52] Ruan Y.-L. (2014). Sucrose metabolism: gateway to diverse carbon use and sugar signaling. *Annu. Rev. Plant Biol.* 65 33–67. 10.1146/annurev-arplant-050213-040251 24579990

[B53] Schmittgen T. D. Livak K. J. (2008). Analyzing real-time PCR data by the comparative CT method. *Nat. Protoc.* 3 1101–1108. 10.1038/nprot.2008.73 18546601

[B54] Sequera-Mutiozabal M. I. Erban A. Kopka J. Atanasov K. E. Bastida J. Fotopoulos V. (2016). Global metabolic profiling of *Arabidopsis* polyamine oxidase 4 (*AtPAO4*) loss-of-function mutants exhibiting delayed dark-induced senescence. *Front. Plant Sci.* 7:173. 10.3389/fpls.2016.00173 26925084 PMC4757743

[B55] Shabala S. Munns R. (2017). *Salinity Stress: Physiological Constraints and Adaptive Mechanisms.* Wallingford: CABI, 24–63. 10.1079/9781780647296.0024

[B56] Sharawy S. M. (2013). Taxonomic relationships of some taxa of subfamily Asclepiadoideae (Apocynaceae) as reflected by morphological variations and polymorphism in seed protein and RAPD electrophoretic profile. *Int. J. Bot.* 9 18–29. 10.3923/ijb.2013.18.29

[B57] Shu K. Qi Y. Chen F. Meng Y. Luo X. Shuai H. (2017). Salt stress represses soybean seed germination by negatively regulating GA biosynthesis while positively mediating ABA biosynthesis. *Front. Plant Sci.* 8:1372. 10.3389/fpls.2017.01372 28848576 PMC5554363

[B58] Simão F. A. Waterhouse R. M. Ioannidis P. Kriventseva E. V. Zdobnov E. M. (2015). BUSCO: assessing genome assembly and annotation completeness with single-copy orthologs. *Bioinformatics* 31 3210–3212. 10.1093/bioinformatics/btv351 26059717

[B59] Singh A. Jha S. K. Bagri J. Pandey G. K. (2015). ABA inducible rice protein phosphatase 2C confers ABA insensitivity and abiotic stress tolerance in *Arabidopsis*. *PLoS One* 10:e0125168. 10.1371/journal.pone.0125168 25886365 PMC4401787

[B60] Soon F.-F. Ng L.-M. Zhou X. E. West G. M. Kovach A. Tan M. H. E. (2014). Molecular mimicry regulates ABA signaling by SnRK2 kinases and PP2C phosphatases. *Science* 335 85–88. 10.1126/science.1215106 22116026 PMC3584687

[B61] Szabados L. Savouré A. (2010). Proline: a multifunctional amino acid. *Trends Plant Sci.* 15 89–97. 10.1016/j.tplants.2009.11.009 20036181

[B62] Székely G. Ábrahám E. Cséplő Á Rigó G. Zsigmond L. Csiszár J. (2008). Duplicated *P5CS* genes of *Arabidopsis* play distinct roles in stress regulation and developmental control of proline biosynthesis. *Plant J.* 53 11–28. 10.1111/j.1365-313X.2007.03318.x 17971042

[B63] Szepesi Á (2020). “Role of metabolites in abiotic stress tolerance,” in *Plant Life Under Changing Environment: Responses and Management*, eds Tripathi D. K. Chauhan D. K. Prasad S. M. Ramawat N. Singh V. P. Sharma S. (Amsterdam: Elsevier), 755–774. 10.1016/B978-0-12-818204-8.00033-3

[B64] Szepesi Á Szőllősi R. (2018). “Chapter 17 - MECHANISM of proline biosynthesis and role of proline metabolism enzymes under environmental stress in plants,” in *Plant Metabolites and Regulation Under Environmental Stress*, eds Ahmad P. Ahanger M. A. Singh V. P. Tripathi D. K. Alam P. Alyemeni M. N. (Cambridge, MA: Academic Press), 337–353. 10.1016/B978-0-12-812689-9.00017-0

[B65] Takahashi T. Kakehi J.-I. (2009). Polyamines: ubiquitous polycations with unique roles in growth and stress responses. *Ann. Bot.* 105 1–6. 10.1093/aob/mcp259 19828463 PMC2794062

[B66] Thalmann M. Santelia D. (2017). Starch as a determinant of plant fitness under abiotic stress. *New Phytol.* 214 943–951. 10.1111/nph.14491 28277621

[B67] van Zelm E. Zhang Y. Testerink C. (2020). Salt tolerance mechanisms of plants. *Annu. Rev. Plant Biol.* 71 403–433. 10.1146/annurev-arplant-050718-100005 32167791

[B68] Verma V. Ravindran P. Kumar P. P. (2016). Plant hormone-mediated regulation of stress responses. *BMC Plant Biol.* 16:86. 10.1186/s12870-016-0771-y PMC4831116 27079791

[B69] Wang J. Lv J. Liu Z. Liu Y. Song J. Ma Y. (2019). Integration of transcriptomics and metabolomics for pepper (*Capsicum annuum* L.) in response to heat stress. *Int. J. Mol. Sci.* 20:5042. 10.3390/ijms20205042 31614571 PMC6829368

[B70] Wang X. Zhou R. Louie G. V. Mühlemann J. K. Bomati E. K. Bowman M. E. (2014). Structural studies of cinnamoyl-CoA reductase and cinnamyl-alcohol dehydrogenase, key enzymes of monolignol biosynthesis. *Plant Cell* 26 3709–3727. 10.1105/tpc.114.127399 25217505 PMC4213152

[B71] Williams C. R. Baccarella A. Parrish J. Z. Kim C. C. (2016). Trimming of sequence reads alters RNA-Seq gene expression estimates. *BMC Bioinformatics* 17:103. 10.1186/s12859-016-0956-2 26911985 PMC4766705

[B72] Wu D. Cai S. Chen M. Ye L. Chen Z. Zhang H. (2013). Tissue metabolic responses to salt stress in wild and cultivated barley. *PLoS One* 8:e55431. 10.1371/journal.pone.0055431 23383190 PMC3561194

[B73] Xie M. Zhang J. Tschaplinski T. J. Tuskan G. A. Chen J.-G. Muchero W. (2018). Regulation of lignin biosynthesis and its role in growth-defense tradeoffs. *Front. Plant Sci.* 9:1427. 10.3389/fpls.2018.01427 30323825 PMC6172325

[B74] Xing B. Gu C. Zhang T. Zhang Q. Yu Q. Jiang J. (2021). Functional study of *BpPP2C1* revealed its role in salt stress in *Betula platyphylla*. *Front. Plant Sci.* 11:617635. 10.3389/fpls.2020.617635 33519877 PMC7841333

[B75] Xue X. Liu A. Hua X. (2009). Proline accumulation and transcriptional regulation of proline biosynthesis and degradation in *Brassica napus*. *BMB Rep.* 42 28–34. 10.5483/BMBRep.2009.42.1.028 19192390

[B76] Yang D. S. Zhang J. Li M. X. Shi L. X. (2017). Metabolomics analysis reveals the salt-tolerant mechanism in *Glycine soja*. *J. Plant Growth Regul.* 36 460–471. 10.1007/s00344-016-9654-6

[B77] Yang Y. Guo Y. (2018). Elucidating the molecular mechanisms mediating plant salt-stress responses. *New Phytol.* 217 523–539. 10.1111/nph.14920 29205383

[B78] Yu Z. Duan X. Luo L. Dai S. Ding Z. Xia G. (2020). How plant hormon mediate salt stress responses. *Trends Plant Sci.* 25 1117–1130. 10.1016/j.tplants.2020.06.008 32675014

[B79] Zhang H. Zhao X. Sun Q. Yan C. Wang J. Yuan C. (2020). Comparative transcriptome analysis reveals molecular defensive mechanism of *Arachis hypogaea* in response to salt stress. *Int. J. Genomics* 2020 1–13. 10.1155/2020/6524093 32190641 PMC7063224

[B80] Zhang J. Yang D. Li M. Shi L. (2016). Metabolic profiles reveal changes in wild and cultivated soybean seedling leaves under salt stress. *PLoS One* 11:e0159622. 10.1371/journal.pone.0159622 27442489 PMC4956222

[B81] Zhang M. Hong L.-Z. Gu M.-F. Wu C.-D. Zhang G. (2020). Transcriptome analyses revealed molecular responses of *Cynanchum auriculatum* leaves to saline stress. *Sci. Rep.* 10 449–459. 10.1038/s41598-019-57219-8 31949203 PMC6965089

[B82] Zhang Y. Butelli E. Alseekh S. Tohge T. Rallapalli G. Luo J. (2015). Multi-level engineering facilitates the production of phenylpropanoid compounds in tomato. *Nat. Commun.* 6 8635–8645. 10.1038/ncomms9635 26497596 PMC4639801

[B83] Zhang Z. Mao C. Shi Z. Kou X. (2017). The amino acid metabolic and carbohydrate metabolic pathway play important roles during salt-stress response in tomato. *Front. Plant Sci.* 8:1231. 10.3389/fpls.2017.01231 28769946 PMC5511834

[B84] Zhao Y. (2018). Essential roles of local auxin biosynthesis in plant development and in adaptation to environmental changes. *Annu. Rev. Plant Biol.* 69 417–435. 10.1146/annurev-arplant-042817-040226 29489397

[B85] Zhu J.-K. (2002). Salt and drought stress signal transduction in plants. *Annu. Rev. Plant Biol.* 53 247–273. 10.1146/annurev.arplant.53.091401.143329 12221975 PMC3128348

[B86] Zörb C. Schmitt S. Neeb A. Karl S. Linder M. Schubert S. (2004). The biochemical reaction of maize (Zea mays L.) to salt stress is characterized by a mitigation of symptoms and not by a specific adaptation. *Plant Sci.* 167 91–100. 10.1016/j.plantsci.2004.03.004

