# Supplementary figures and images for: Integrated de novo Analysis of Transcriptional and Metabolic Variations in Salt-Treated Solenostemma argel Desert Plants
Source: Front Plant Sci. 2021 Nov 19;12:744699. doi: 10.3389/fpls.2021.744699 (PMC8640078; doi:10.3389/fpls.2021.744699)

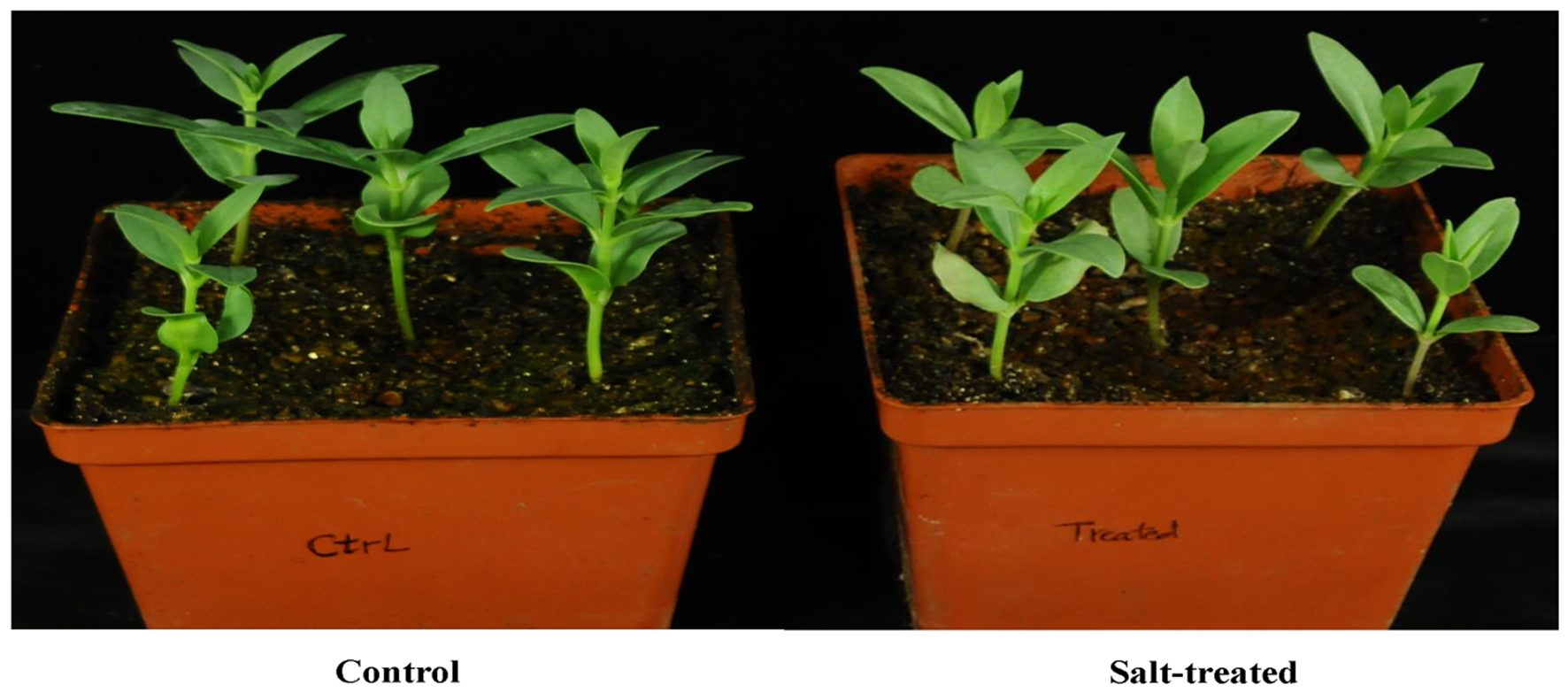

Supplement: Supplementary Figure 1 — Salinity influence on phenotypic characteristics of S. argel seedlings after 3-days treatment in a greenhouse. Control: control seedlings, treated: seedlings treated with 500 mM NaCl for 3 days. [file Image_1.TIFF]
