# Supplementary material for: Integrated de novo Analysis of Transcriptional and Metabolic Variations in Salt-Treated Solenostemma argel Desert Plants
Source: Front Plant Sci. 2021 Nov 19;12:744699. doi: 10.3389/fpls.2021.744699 (PMC8640078; doi:10.3389/fpls.2021.744699)
Supplement: Supplementary file 2 [file Table_1.docx]

Supplementary Table S1. Forward and reverse primer sequences were used for qRT-PCR analysis of 16 genes differentially expressed in roots and leaves of *S*. *argel* plants.

| No |  | KEGG No | Gene name | Foreword | Reverse |
| --- | --- | --- | --- | --- | --- |
| 1 | Roots | K00827 | AGXT2 | CATTCCTCCACCCTACGACG | AGGGCTGAGAAACTCTTGACG |
| 2 |  | K01188 | BGLU | CCCCGGAAGATGCTCTCTTTT | TGTGTGCTACCAGGTAAGGC |
| 3 |  | K01850 | CM1 | ACTATGGGAAATTTGTTGCTGAAG | AGCTCGAATTGCTGAGGAAA |
| 4 |  | K05280 | F3′H | TGCAAGGGATGCTAGATGAGT | ACCCTTGCAAATCGAATCTACT |
| 5 |  | K11816 | YUCCA | TGGATTGTACTGCGCTGGTT | TCAGGGATGGTGATCGAGGA |
| 6 |  | K12657 | P5CS | AAGGTCTGCTCACGACCAAGT | TTCGCGATCAGCTCCAGCTTT |
| 7 | Leaves | K01626 | aroF | TTACAGTGGACCATCCCGTG | TCATAAGGCAGGAGCAAGCA |
| 8 |  | K00128 | ALDH2 | GCAGCAGCAGAAGGGTTCAT | CTGGATCCAAGCTTGAAGGACT |
| 9 |  | K00815 | TAT | TCCAGACTCGTTGGATCCGT | TGCCAGGATTAGCAGTTGGT |
| 10 |  | K01915 | GLUL | GACCAGCCTCGAACATGGAT | GGTTTCCAGAGGATGGTGGT |
| 11 |  | K09753 | CCR | GCCAATTCTGTGCAGGCTTA | GGAAGCAGAGGGAGTTTCGT |
| 12 |  | K00083 | CAD | AACAGTGTACTGCCCGATGA | CGTGACCCAAACCTCCAAGA |
| 13 | Roots and Leaves | K13832 | DHQ-SDH | TGTGAACCCAGTGATGTAGCA | GATGTGAAACCGCCGTCAAC |
| 14 |  | K00430 | PRX | GCTGCCAGAGAGGCTGTATTT | ATTCGCGGTTAGACCATCTCG |
| 15 |  | K00826 | ilvE | GTGTTGCCTCAGTGGGAAGT | TTACCACTTCGAATCCCGGC |
| 16 |  | K05909 | Lac | AGCATATTGTGCCCTGTGGAA | TGCAAAACAGGAAACTAACTGCT |
| 17 |  |  | BnActin7 | GGAACTGGAATGGTGAAGGCTG | GTCTTTTTGACCCATCCCAAC |
